# Developmental Exposure to a Commercial PBDE Mixture: Effects on Protein Networks in the Cerebellum and Hippocampus of Rats

**DOI:** 10.1289/ehp.1408504

**Published:** 2014-12-19

**Authors:** Prasada Rao S. Kodavanti, Joyce E. Royland, Cristina Osorio, Witold M. Winnik, Pedro Ortiz, Lei Lei, Ram Ramabhadran, Oscar Alzate

**Affiliations:** 1 Neurotoxicology Branch, and; 2 Genetic and Cellular Toxicology Branch, Office of Research and Development (ORD), U.S. Environmental Protection Agency (EPA), Research Triangle Park, North Carolina, USA; 3 Systems Proteomics Center, and; 4 Program in Molecular Biology and Biotechnology, University of North Carolina at Chapel Hill, Chapel Hill, North Carolina, USA; 5 NHEERL Proteomics Research Core, ORD, U.S. EPA, Research Triangle Park, North Carolina, USA; 6 Department of Cellular and Developmental Biology, University of North Carolina at Chapel Hill, Chapel Hill, North Carolina, USA.; * These authors contributed equally to this work.

## Abstract

Background: Polybrominated diphenyl ethers (PBDEs) are structurally similar to polychlorinated biphenyls (PCBs) and have both central (learning and memory deficits) and peripheral (motor dysfunction) neurotoxic effects at concentrations/doses similar to those of PCBs. The cellular and molecular mechanisms for these neurotoxic effects are not fully understood; however, several studies have shown that PBDEs affect thyroid hormones, cause oxidative stress, and disrupt Ca^2+^-mediated signal transduction. Changes in these signal transduction pathways can lead to differential gene regulation with subsequent changes in protein expression, which can affect the development and function of the nervous system.

Objective: In this study, we examined the protein expression profiles in the rat cerebellum and hippocampus following developmental exposure to a commercial PBDE mixture, DE-71.

Methods: Pregnant Long-Evans rats were dosed perinatally with 0 or 30.6 mg/kg/day of DE-71 from gestation day 6 through sampling on postnatal day 14. Proteins from the cerebellum and hippocampus were extracted, expression differences were detected by two-dimensional difference gel electrophoresis, and proteins were identified by tandem mass spectrometry. Protein network interaction analysis was performed using Ingenuity® Pathway Analysis, and the proteins of interest were validated by Western blotting.

Results: Four proteins were significantly differentially expressed in the cerebellum following DE-71 exposure, whereas 70 proteins were significantly differentially expressed in the hippocampus. Of these proteins, 4 from the cerebellum and 47 from the hippocampus, identifiable by mass spectrometry, were found to have roles in mitochondrial energy metabolism, oxidative stress, apoptosis, calcium signaling, and growth of the nervous system.

Conclusions: Results suggest that changes in energy metabolism and processes related to neuroplasticity and growth may be involved in the developmental neurotoxicity of PBDEs.

Citation: Kodavanti PR, Royland JE, Osorio C, Winnik WM, Ortiz P, Lei L, Ramabhadran R, Alzate O. 2015. Developmental exposure to a commercial PBDE mixture: effects on protein networks in the cerebellum and hippocampus of rats. Environ Health Perspect 123:428–436; http://dx.doi.org/10.1289/ehp.1408504

## Introduction

Polybrominated diphenyl ethers (PBDEs) have been used as flame retardants in domestic and industrial applications, including computers, television sets, mobile telephones, furniture, textiles, insulation boards, mattresses, and upholstery furnishings (Alaee et al. 2003). Like polychlorinated biphenyls (PCBs), PBDEs are structurally similar synthetic chemicals composed of two phenyl rings linked by oxygen (thus the designation as “ethers”; see Supplemental Material, Figure S1). PBDEs are ubiquitous in the environment, where they bioaccumulate, becoming toxic to animals and humans (Kodavanti et al. 2008). Levels of PBDEs have been reported to be increasing in some parts of the environment, in human blood, and in milk (McDonald 2005).

PBDEs are typically produced for industrial use at three different levels of bromination, that is, penta-, octa-, and decabrominated diphenyl ether mixtures (La Guardia et al. 2006; World Health Organization 1994). Commercially available PBDE products are not single compounds or even single congeners but rather a mixture of congeners. The commercial PBDE mixture DE-71 consists of > 20 different congeners. Its primary constituents include 2,2´,4,4´-tetrabromodiphenyl ether (PBDE 47, ~ 38%) and 2,2´,4,4´,5-pentabromodiphenyl ether (PBDE 99, ~ 49%). Collectively, these two congeners account for approximately 87% (wt/wt) of the DE-71 mixture (La Guardia et al. 2006). In the United States, PBDE 47 and PBDE 99 are the two most predominant congeners detected in human milk, serum, and whole blood (Schecter et al. 2005). Like other lipophilic compounds, PBDEs readily cross the placenta into the fetus and accumulate in milk resulting in infant exposure during lactation, providing an opportunity for PBDEs to interfere with developmental processes (Kodavanti et al. 2010; Mazdai et al. 2003).

Several studies have shown that PBDE exposure results in alterations in spontaneous behavior and in reduced learning and memory in mice (Viberg et al. 2003a, 2003b, 2004). These effects were similar to those seen after neonatal exposure to the structurally related chemicals, the PCBs (Eriksson and Fredriksson 1996). Rice et al. (2007) reported developmental delays in the acquisition of the palpebral reflex following repeated neonatal exposure to PBDE 209, along with changes in circulating levels of thyroxine (T_4_). However, Gee and Moser (2008) observed that mice exposed to PBDE 47 on postnatal day (PND) 10 displayed a delayed ontogeny of neuromotor functional end points as well as adult hyperactivity. Considering critical neurodevelopment effects including habituation response for PBDEs, the U.S. Environmental Protection Agency’s (EPA) derived reference dose (RfD) values were 0.1, 0.1, and 0.2 μg/kg/day, respectively, for PBDEs 47, 99, and 153 (U.S. EPA 2008a, 2008b, 2008c).

In a previous study using the same cohort of animals, we found that DE-71 was associated with a significant decrease in circulating T_4_ levels and accumulation of PBDE congeners in various tissues, including the brain (Kodavanti et al. 2010). This suggests that PBDEs cross the blood–brain barrier, potentially causing changes in neurobehavioral parameters (Kodavanti et al. 2010). In addition, we previously reported that PBDEs, like PCBs, affect intracellular signaling pathways including calcium homeostasis, mitogen-activated protein kinase, and translocation of protein kinase C (PKC) (Fan et al. 2010; Kodavanti et al. 2005). All of these signaling pathways are known to be associated with the development of the nervous system and involved in learning and memory (Kater and Mills 1991; Lein et al. 2007). Perturbations of such signal transduction pathways in the brain could affect gene regulation and alter protein expression, which might have an ultimate effect on nervous system growth and function. In the present study, we used two-dimensional difference gel electrophoresis (2D DIGE)–based quantitative intact proteomics (QIP) to examine the protein expression profiles in the cerebellum and hippocampus of rats perinatally exposed to DE-71.

## Materials and Methods

*Animals and chemical exposure*. Timed-pregnant Long-Evans rats (body weight ~ 230 g; Charles River Laboratories, Portage, MI) were obtained on gestational day (GD) 3 (day of insemination is GD0) and were housed individually in standard polycarbonate plastic cages containing heat-treated pine shavings as bedding and maintained under regulated temperature (21 ± 2°C), relative humidity (50 ± 10%), and a 12-hr light/dark cycle. Food (Purina Formulab Diet 5008 through lactation and Laboratory Rodent Diet 5001 postweaning) and water were provided *ad libitum* (Kodavanti et al. 2010). The protocols and the use of animals in all experiments were approved by the animal care and use committee of the U.S. EPA National Health and Environmental Effects Research Laboratory, and the animals were treated humanely and with regard for alleviation of suffering.

The commercial PBDE mixture DE-71 (lot no. 1550OI18A) was a gift from the Great Lakes Chemical Corporation (El Dorado, AR). The presence of impurities, including brominated biphenyls, dioxins, and furans, has been reported elsewhere (Hanari et al. 2006). The dose of DE-71 (30.6 mg/kg/day in corn oil) was selected to match, on a molar basis, the doses of Aroclor-1254 used in previous studies for which we have extensive information, both *in vitro* and *in vivo* (reviewed by Kodavanti 2005). The use of these previously described doses allows us to compare the effects of these two structurally related groups of chemicals. Dams (*n* = 15/dose group) were weighed and administered DE-71 in corn oil or corn oil alone (2 mL/kg body weight) by oral gavage daily (between 0800 and 1000 hours) from GD6 through PND14, except on PND0 when the dams were not disturbed. Dams delivering a litter of 10–15 pups were used in the study. On PND4, litters were culled to 8 pups with a minimum of 5 males.

The reproductive outcomes, decreases in circulating T_4_ levels, assignment of rats for different measures, general health, and development of the rats used in these studies, as well as biological relevance of the highest dose used in this study in terms of human levels, have been previously reported (Kodavanti et al. 2010). No changes were found in maternal or pup body weights or pup motor activity. For the present study, three males per treatment group, each from a different litter were sacrificed by guillotine on PND14; cerebella and hippocampi were dissected, quickly frozen on dry ice, and stored at –80°C until further processing. Dissections were carried out in a necropsy room at 1000 hours in the animal facility following morning dosing and weighing protocols.

*Protein extraction*. Preparation of samples for proteomic analysis was as previously described (Kodavanti et al. 2011). Briefly, each tissue fraction (approximately 40 mg) was suspended in 200 μL of lysis buffer [8 M urea, 2 M thiourea, 4% CHAPS, 20 mM Tris pH 7.5, supplemented with protease inhibitor Complete® (Pierce, Rockford, IL) and phosphatase inhibitor, NaVO_4_ (Sigma, St. Louis, MO)]. Protein lysates were prepared using a 2D-Clean Up Kit (GE Healthcare, Piscataway, NJ), and the resulting pellet was resuspended in focusing buffer (8 M urea, 4% CHAPS, 30 mM Tris-HCl, pH 8.5). Protein concentration was determined with a GE Healthcare 2D-Quant kit as directed by the manufacturer.

*2D DIGE analysis*. Differential protein expression was determined using 2D DIGE–based QIP (Alban et al. 2003; Friedman et al. 2004) following a three-dye protocol that allows simultaneous labeling of an internal control and brain samples from exposed and unexposed rats. This protocol has been shown to greatly reduce the variability inherent in 2D-gel protein analysis. All experimental procedures were as previously described (Kodavanti et al. 2011). A total of three brains each from control and exposed rats were used. Briefly, 120 μg of total protein from each exposed sample and from each control sample was labeled with 400 pmol of the cyanine dyes Cy5 and Cy3 (GE Healthcare), respectively. The internal control was created by pooling 60 μg from control and exposed samples followed by labeling with 400 pmol Cy2.

Isoelectric protein separation of labeled samples (*n* = 3 animals/group) was performed using immobilized pH gradient strips [13 cm, pI (isoelectric point) range, 3–10; GE Healthcare]. Protein separation in the second dimension was done with SDS (12%)-PAGE gels (4% stacking) casted on low fluorescence glass plates (13 cm; GE Healthcare). Each control sample was run against each exposed sample for a total of nine gels, improving statistical power. The resulting gels were scanned using a Typhoon 9410 imaging system (GE Healthcare) and then were fixed and stained with colloidal Coomassie for further processing.

*Protein identification*. Protein identification was performed as previously described (Winnik et al. 2012). Briefly, spots with statistically significant expression changes as determined by DeCyder 2D software (GE Healthcare) and of sufficient amount of protein were excised from the gels with the Ettan Spot Picker (GE Healthcare). In-gel protein digestion was carried out using modified trypsin, and matrix-assisted laser desorption ionization (MALDI) tandem mass spectrometry (MS/MS) data were acquired with either a 4700 or a 4800 Proteomics Analyzer MALDI tandem time-of-flight (TOF/TOF) mass spectrometer (Applied Biosystems). The proteins were identified by a combination of peptide mass fingerprinting and the sequence-tag approach (DeKroon et al. 2011). The peptide mass fingerprinting and sequence tag data were evaluated with Mascot scores (Mascot® Search Engine; Matrix Science; http://www.matrixscience.com/) or “Aldente” online proteomic software (Winnik et al. 2012). Keratin and trypsin autolysis contaminant peaks were identified and excluded from the MS/MS analysis. This step was followed by MALDI MS/MS sequencing, and the final protein identification was performed using Protein Pilot 3.0 software (Applied Biosystems) and the Mascot® Search Engine, searching against the rat species database within the nonredundant National Center for Biotechnology Information database (NCBI nr) or the SwissProt database (ExPasy Bioinformatics Resource Portal 2013). Finally, mass-selected MALDI MS/MS spectra acquisition was performed to further increase protein sequence coverage. Proteins were identified based on at least two unique MS/MS peptide sequences with confidence scores > 95% (corresponding to the overall minimum protein identification confidence level of 99.75%) based on Protein Pilot 3.0 peptide and protein percent confidence score criteria. Alternatively, an MS/MS-based MASCOT protein score of ≥ 40 was considered acceptable (Winnik et al. 2012).

*Western blot analysis*. Each sample was prepared as described previously (Kodavanti et al. 2011). Briefly, 200 mg of tissue was sonicated in 200 μL RIPA buffer (Sigma) with Complete® protease inhibitor cocktail (Roche) and centrifuged at approximately 20,000 × *g* for 10 min at 5°C. The supernatant was collected and protein content was determined with a 2D-Quant kit (GE Healthcare). A 10-μg aliquot of total protein was separated on 10% SDS-PAGE gels, the proteins were transferred to polyvinylidene difluoride membranes for 16 hr in a cold room, and the membranes were probed with monoclonal antibodies from Abcam, (Cambridge, MA) against anti-protein disulfide isomerase A3 (PDIA3; ab10287) and α-tubulin (ab7291) as an internal loading control (Alban et al. 2003). We chose PDIA3 [also called endoplasmic reticulum resident protein 57 (ERP57)] for Western blot analysis to validate the 2D-gel data set because it possesses multiple functional roles in central nervous system physiology that were impacted by gestational and lactational exposure to DE-71.

*Protein functional analysis*. We used the NCBI databases (http://www.ncbi.nlm.nih.gov/) to research protein functional ontology. The resulting information was used to separate the differentially expressed proteins into functional groups. Follow-up analysis with Ingenuity® Pathway Analysis (IPA) software v9.0-3210 (Ingenuity _ENREF_31; http://www.qiagen.com/ingenuity) provided additional correlations between identified proteins and was used to generate functional networks as described previously (Kodavanti et al. 2011). Networks were ranked by their scores, which are based on a *p*-value calculation on the probability that network proteins are part of the network by random chance and are equal to the negative exponent of this calculation. For example, a score of 25 reflects highly relevant networks with *p*-values of 10^–25^. Canonical overlays had *p*-values ≤ 0.05.

*Statistics*. The digital images from the Typhoon system were analyzed with DeCyder 2D software to determine changes in protein levels across gels and for pairwise comparisons of individual Cy3- and Cy5-labeled samples (Friedman et al. 2004). Pair-wise comparisons of each PND14 control and the corresponding DE-71 sample, along with the pooled internal standard present on each gel, was performed using the DeCyder differential in-gel analysis module. We used 2 SDs from the mean volume ratio (95th percentile confidence interval) as a threshold to determine the levels of significance for a given set of samples. Statistical analysis and gel-to-gel comparisons were performed with Decyder’s Biological Variation Analysis module (DeKroon et al. 2011). Data were log2 transformed, and protein changes between control and DE-71–exposed rats were analyzed using Student’s *t*-test (*p* < 0.05). Fold changes calculated as the ratio of the treated to the control were derived on the mean expression changes determined. Significance for Western analysis was determined by *t*-test at *p* ≤ 0.05 (Microsoft Excel 2013; Microsoft Corp., Redmond, WA).

## Results

**Figure 1:**
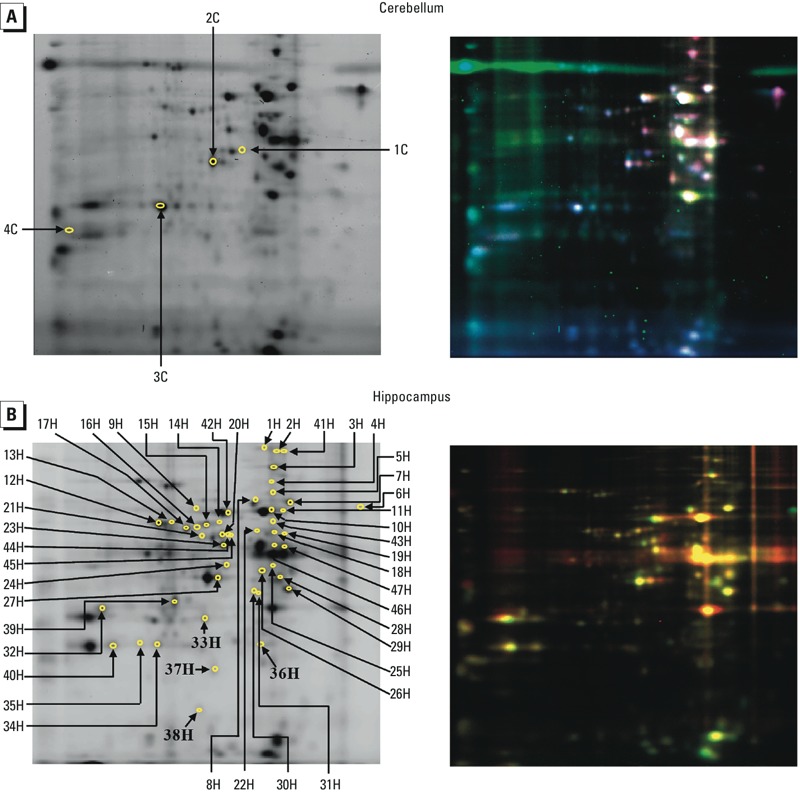
Representative 2D DIGE gels from the cerebellum (*A*) and hippocampus (*B*) showing proteins whose expression was different in rats developmentally exposed to DE‑71 compared with controls. (Left) The numbered protein spots represent differentially expressed proteins identified in Supplemental Material, Table S1. The arrows point to proteins that were altered in tissue from DE‑71–exposed rats as determined by DeCyder 2D software; 4 proteins were altered in the cerebellum, and 70 proteins were altered in the hippocampus. (Right) Images illustrate protein spots fluorescently labeled with Cy2, Cy3, or Cy5. DeCyder 2D software corrected for variation in spot colors occuring with individual gel staining. Red represents proteins with higher expression in the treated sample; green indicates proteins with higher levels in the control sample; and white or yellow represents proteins with no difference in expression level between control and DE‑71–exposed rats. *n *= 3 rat brains per treatment.

**Figure 2:**
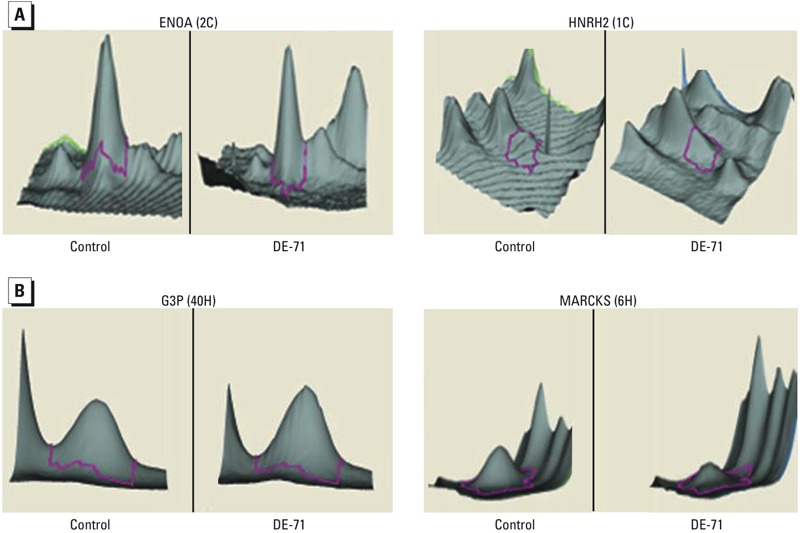
Three-dimensional visualization of protein spots differentially expressed in the cerebellum (*A*) and hippocampus (*B*) of DE‑71–exposed rats compared with controls. Examples show how high [*A,* left; ENOA (2C)] and low [A, right; HNRH2 (1C)] expression changes or up- [*B*, left; G3P (40H)] and down- [*B*, right; MARCKS (6H)] regulation were identified by the DeCyder 2D software. Details for the proteins are provided in Supplemental Material, Table S1.

*Differential protein expression analysis*. Perinatal DE-71 exposure had a significant impact on cerebellar and hippocampal protein expression at PND14. Figure 1 shows representative examples of gels stained with three dyes (as scanned by the Typhoon 9410 imaging system) identifying protein spots that are significantly different in the cerebellum (Figure 1A) and hippocampus (Figure 1B) of DE-71–exposed animals compared with controls. These images, analyzed by DeCyder 2D software, resulted in three-dimensional (3D) images representing changes in protein levels in the cerebellum (Figure 2A) and hippocampus (Figure 2B). Analysis of the 3D images identified 4 and 70 protein spots as differentially expressed in the cerebellum and hippocampus, respectively.

Proteins available in amounts sufficient for downstream procedures were identified by MALDI TOF/TOF MS analysis. This included all 4 of the cerebellar protein spots and 47 of the 70 differentially expressed hippocampal protein spots (*p* < 0.05; Figure 1). Protein identification features, including accession number, isoelectric point, molecular weight, and Mascot protein score, are presented in Supplemental Material, Table S1. Seven of the identified hippocampal proteins had two posttranslational modifications [albumin (ALBU), glial fibrillary acidic protein (GFAP), dihydropyrimidinase-related protein 2 (DPYL2), DPYL5, heat shock protein 74 (HSP74), creatinine kinase B-type (KCRB), and tubulin alpha-1B (TBA1B)], one had two isoforms [glyceraldehyde-3-phosphate dehydrogenase (G3P)], and one protein (DPYL3) was identified in 4 isoforms; thus, the final count for identified hippocampal proteins was 35. All of the cerebellar proteins (4/4) (see Supplemental Material, Table S2) and approximately 94% (66/70) of the hippocampal proteins (see Supplemental Material, Table S3) affected were up-regulated by DE-71 exposure.

*Protein functional analysis*. Using the NCBI and Swiss-Prot databases, we ascertained individual protein functions based on the specific accession numbers determined from protein identification software (DeCyder 2D software). Proteins were then grouped into larger functional categories by correlating changes in expression with possible functional response to and consequences of DE-71 exposure. Differentially expressed proteins in the cerebellum were related to growth functions associated with carbohydrate metabolism [alpha-enolase (ENOA) and fructose-bisphosphate aldolase C (ALDOC)] and RNA processing [heterogeneous nuclear ribonucleoprotein (HNR) H2 and heterogeneous nuclear ribonucleoprotein A3 (ROA3)] (see Supplemental Material, Table S2). Growth functions were also prominently displayed in the identified hippocampal proteins, with 18 of the 35 identified proteins falling into this general category. Proteins with roles in cytoskeleton assembly [e.g., septin-5 (SEPT5), myristoylated alanine-rich-C-kinase (MARCKS)], energy metabolism (e.g., KCRB), neurotransmission [e.g., glutamine synthetase (GLNA)], and cell division [transitional endoplasmic reticulum ATPase (TERA)], as well as proteins with roles in common with those identified in the cerebellum, such as carbohydrate metabolism [ENOA and gamma-enolase (ENOG)] and RNA processing (HNRH1), are included in this group. The next major functional group in the hippocampus is related to proteins linked to the stress response and is indicative of the adverse effects of DE-71 exposure. Protein markers of neuronal death (GFAP) and microglial cell activation (ALBU); heat shock proteins that act as protein chaperones (e.g., HSP74 and HSP7C); and PDIA3, a protein involved in redox metabolism, were differentially expressed in DE-71–exposed rat hippocampi. Several proteins with roles in neuronal plasticity, possibly in response to damage, were also identified. For example, proteins involved in neuronal development [e.g., DPYL2, DPYL3, DPYL4, dynactin subunit 2 (DCTN2)] and synaptogenesis (DCTN2) were up-regulated. The final functional group is related to protein chemistry and is composed of proteins with roles in protein catabolism [proteosome subunit alpha type-1 (PSA1)], complex assembly [glucose regulated protein 78 kDa (GRP78)], formation of secondary structure [T-complex protein 1 subunit (TCP) epsilon and gamma], and synthesis [eukaryotic translation initiation factor 4B (Q5RKG9)].

**Figure 3:**
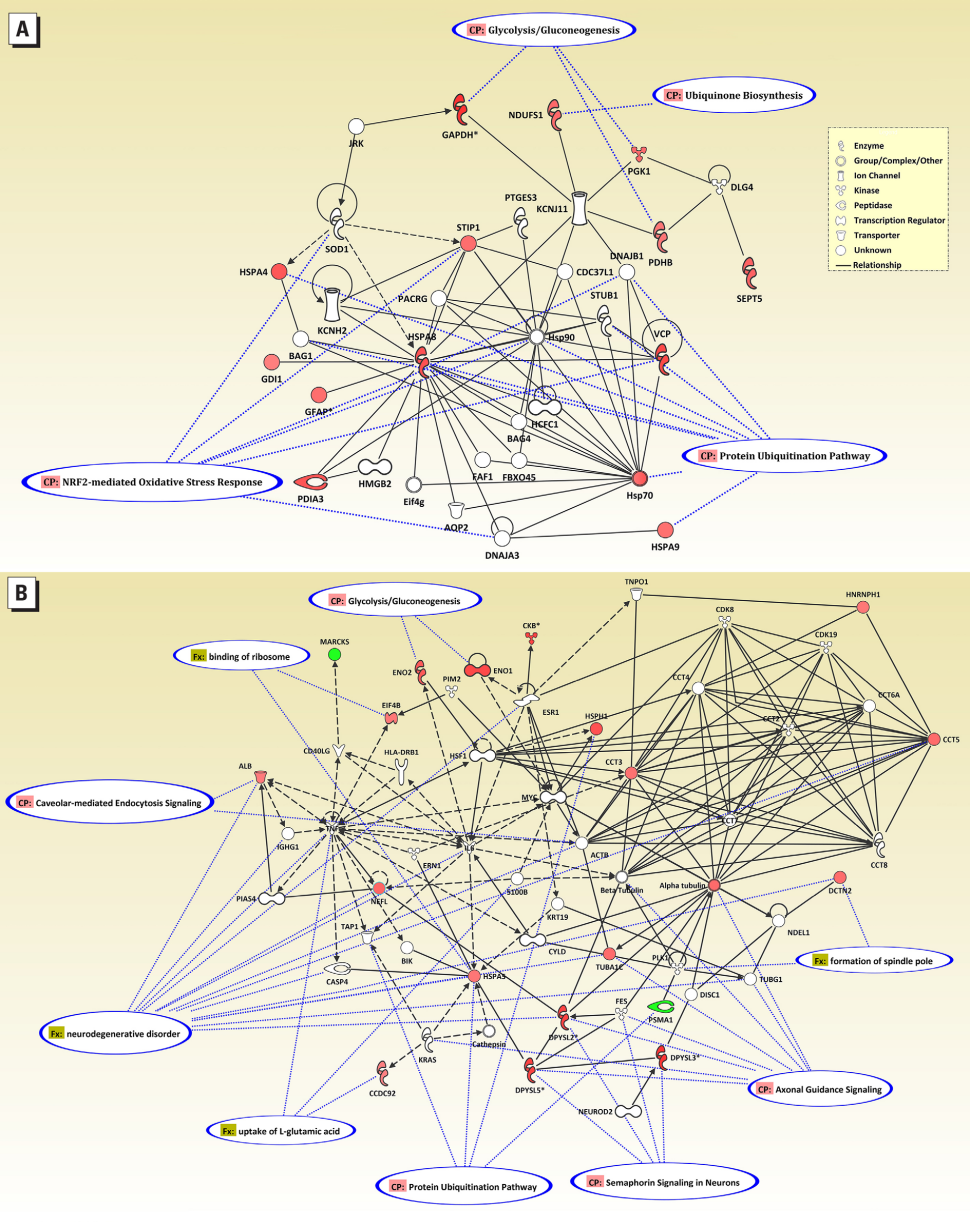
Ingenuity® Pathway Analysis showing functional correlates of proteins that were affected in the hippocampus of rats developmentally exposed to DE‑71. (*A*) The most highly correlated network (score of 25). (*B*) Merged network of the next three highest ranked networks (scores of 17, 9, and 2). Symbols for differentially expressed proteins are in shades of red, with color intensity related to level of change, and symbols for networked proteins (from the Ingenuity database) are white. Associated canonical overlays are indicated by blue-outlined ovals and dotted blue lines.

The results of the IPA performed on the identified hippocampal proteins agreed closely with the functional ontology results. The network in Figure 3A represents the top network, with a score of 25 (*p* < 10^–25^). Fourteen of the 35 identified proteins fell within this network, which included proteins involved in post-translation modification, protein folding, and drug metabolism. Canonical overlays highlighted the nuclear factor erythroid 2-related factor (Nrf2)-mediated oxidative stress, glucose metabolism, and protein modification pathways. The network in Figure 3B shows the results from merging three of the remaining top networks with scores of 17, 9, and 2 (*p* < 10^–17^, 10^–9^, and 10^–2^, respectively), which included proteins with roles in cellular function and maintenance, cellular assembly and organization, and nervous system development and function. The remaining DE-71–affected hippocampal proteins are in this network. For example, the enolases alpha-enolase (ENOA) and gamma-enolase (ENOG) participate in glucose metabolism, and eukaryotic translation initiation factor 4B, which binds ribosomes and functions in synthetic processes, contributes to cell growth and maintenance. Tubulin and the dihydropyrimidinase-related proteins (DPYL2, DPYL3, and DPYL5) have roles in cytoskeleton and axonal growth and contribute to both cell organization and neuronal development. Similar functions can be identified for the other proteins (see Supplemental Material, Table S3). Canonical and functional overlays include protein modification and glucose metabolism as in network I, but they also include signaling and neuronal functions.

*Western blot confirmation*. We validated 2D DIGE results by Western blot analysis of PDIA3 (also called as ERP57) in the hippocampus of control (0 mg/kg) and DE-71–exposed (30.6 mg/kg) rats as shown in Supplemental Material, Figure S2A. The mean fold-change (± SE) was 1.78 ± 0.28 in DE-71–exposed rats compared with controls; data were from three independent experiments (*n* = 3 per treatment). These data closely match that of the 2D-gel experiment (1.67-fold increase; see Supplemental Material, Table S3). Supplemental Material, Figure S2B, shows the representative protein bands for PDIA3 and the housekeeping protein, α-tubulin (used as an internal control), found in the hippocampus of control and treated rats.

## Discussion

There is growing evidence that PBDEs have an adverse impact on neurobehavioral and functional development. Epidemiological studies have shown associations between prenatal PBDE exposure and reduced development in children, including both psychomotor development and Full-Scale IQ performance (Eskenazi et al. 2013; Herbstman et al. 2010). These epidemiological studies are supported by animal studies in which PBDE exposure delayed the ontogeny of neuromotor function, resulting in hyperactivity in adult mice (Gee and Moser 2008). Viberg et al. (2003a, 2003b, 2004) reported persistent aberrations in spontaneous behavior and habituation capability in mice after a single developmental exposure (PND10) with PBDEs 99, 153, and 209. A study in our laboratory on developmental exposure to DE-71 found that accumulation of PBDE congeners in various tissues, including the brain, were associated with subtle changes in parameters of neurobehavior and decreased circulating T_4_ levels (Kodavanti et al. 2010). Perturbed thyroid hormone homeostasis, altered cell signaling including calcium homeostasis, and neurotransmitter changes have been postulated as critical events leading to the adverse neuronal effects of persistent organic pollutants such as PBDEs (Kodavanti 2005). In the present study, we examined changes in protein profiles in the cerebellum and hippocampus following developmental exposure to DE-71. The results indicated that perinatal exposure to DE-71 altered the expression of proteins associated with key metabolic pathways involved in cell growth, protein metabolism, neuronal plasticity, and stress responses (Figure 3; see also Supplemental Material, Tables S2 and S3). The effect of DE-71, as measured by the number of altered proteins, was greater in the hippocampus than in the cerebellum, most likely due to ontogenetic differences in the two brain areas during time of exposure (GD6–PND14). In contrast to most brain regions, both the hippocampus and cerebellum largely develop postnatally (Buell et al. 1977). However, postnatal hippocampal neurogenesis continues at some level for up to 3 months of age in the rat, whereas cerebellar neurogenesis begins to decline at about 6 days post birth (Altman and Das 1965).

Maintaining normal cell function requires an adequate energy supply, especially during growth and under conditions of stress. We found differential expression of multiple proteins with roles in energy production in tissue of rats developmentally exposed to DE-71. Glycolysis seemed to be mainly affected, suggesting greater changes in the levels of cytoplasmic proteins. ENOA, a component of glycolysis that generates ATP through high energy intermediates (Wold 1971), was significantly up-regulated both in the cerebellum and hippocampus. Alm et al. (2006) reported a similar increase in the mouse hippocampus following a single oral dose of PBDE 99 on PND10. In the same pathway, ALDOC was up-regulated in the cerebellum, whereas G3P and phosphoglycerate kinase 1 (PGK1) were up-regulated in the hippocampus (see Supplemental Material, Tables S2 and S3). ALDOC splits fructose 1,6-bisphosphate into dihydroxyacetone phosphate and G3P. G3P and PGK1 catalyze the sixth and seventh steps of glycolysis, generating ATP. G3P is also the precursor to pyruvate which feeds into the Krebs cycle. This is particularly important in the brain, which uses primarily mitochondrial oxidative phosphorylation as its source of ATP (Hall et al. 2012). IPA identified gluconeogenesis/glycolysis to be one of the highest ranked altered pathways in this data set (Figure 3). Additional proteins related to glycolysis (pyruvate dehydrogenase E1; ODPB), the Krebs cycle (malate dehydrogenase; MDHC), electron transport (NADH-ubiquinone oxidoreductase; NDUS1), and energy transduction (KCRB) were differentially expressed in the hippocampus. KCRB is specific for brain tissue and plays a major role in generating ATP under conditions of high energy demand (Mahadevan et al. 1984). Although creatine kinase is widely regarded as a soluble enzyme, the brain isoform is associated with synaptic plasma membrane (Lim et al. 1983). It has been shown to be regulated by thyroid hormones, with increased serum KRCB levels being associated with hypothyroidism (Beyer et al. 1998). Developmental DE-71 exposure is known to cause hypothyroidism by decreasing circulating T_4_ levels (Kodavanti et al. 2010), and this effect could play a role in the alterations of KRCB seen in the hippocampus. Mukherjee et al. (2013) recently reported that PBDE-47 altered several proteins related to energy metabolism, including ENOA. PBDE-154 has been shown to deplete mitochondrial ATP by interacting with the inner mitochondrial membrane, inhibiting electron transport, and reducing the membrane potential (Pereira et al. 2014), and PBDE-49 inhibited electron transport complexes IV and V in brain mitochondria (Napoli et al. 2013). These data highlight the critical role of energy metabolism in DE-71’s mode of action.

In addition to ENOA, proteins related to nucleotide metabolism were also altered both in the cerebellum and hippocampus. HNRH2 and HNRH1 were up-regulated in the cerebellum and hippocampus, respectively. In addition, ROA3 was increased in the cerebellum and heterogeneous nuclear ribonucleoprotein K (HNRHK) was increased in the hippocampus by DE-71. These proteins have multiple functions in the processing of heterogeneous nuclear RNAs into mature mRNAs and can act as *trans*-factors in regulating gene expression (Chaudhury et al. 2010).

In another growth-related function, several hippocampal proteins related to cytoskeleton structure and axonogenesis were changed by DE-71 (see Supplemental Material, Table S3). Tubulin alpha-1B chain (TBA1B), tubulin alpha-1C (TBA1C), SEPT5, and neurofilament light polypeptide (NFL) were up-regulated, whereas MARCKS was down-regulated. Along with actins, tubulins are abundant cytoskeletal proteins that support diverse cellular processes, including microtubule and microfilament structure and function (Lundin et al. 2010). Septins are GTP-binding proteins with roles in vesicle trafficking, apoptosis, remodeling of the cytoskeleton, neurodegeneration, and neoplasia (Hall et al. 2005). MARCKS protein has been implicated in actin cytoskeletal rearrangement in response to extracellular stimuli (Eun et al. 2006). Mukherjee et al. (2013) reported a similar increase in cytoskeletal proteins (tubulin beta chain and actin) following BDE-47 exposure in the snail, *Crepidula onyx*. In neural stem/progenitor cells, BDE-209 and/or BDE-47 decreased the expression of cytoskeletal proteins such as cofilin-1 and vimentin (Song et al. 2014). Together, these data suggest perturbed neuronal processes, either as a disruption of normal neurite outgrowth or possibly as a repair response to the DE-71–induced neurotoxic effects, in agreement with the study of Viberg and Eriksson (2011) in which changes in proteins involved in maturation of brain, neuronal growth, and synaptogenesis were associated with changes in learning and memory resulting from PBDE exposure.

Further evidence of an impact on neurogenesis can be seen with the effect of DE-71 on the dihydropyrimidinase-related proteins (DPYL2, DPYL3, and DPYL5) for which multiple isoforms were affected. These proteins are involved in facilitating neuronal growth cone migration and promoting microtubule assembly, and they have a role in synaptic signaling. They are required for signaling by the cell-adhesion semaphorin proteins (Suzuki et al. 2003). Dihydropyrmidinase-related proteins have been targets for oxidative stress in brains affected by Alzheimer’s disease (Castegna et al. 2002), and both DPYL proteins and their companion semaphorins have been reported to be up-regulated in response to nervous system injury (Murphey et al. 1999). In the present study, IPA identified axonal guidance, semaphorin signaling, and neurodegenerative process as important pathways in DE-71 neurotoxicity (Figure 3). Also involved in synaptic plasticity, the dynactin protein DCTN2 was up-regulated by DE-71. Dynactin is necessary for stabilization of the synapse and for dendritic arborization during development (Eaton et al. 2002; Murphey et al. 1999). Dingemans et al. (2007) observed that PBDE-47 exposure reduced long-term potentiation together with changes in postsynaptic proteins involved in synaptic plasticity in the mouse hippocampus.

Multiple proteins up-regulated in the present study suggest possible pathways for PBDE-induced neurotoxicity. For example, GFAP, a marker of neuronal damage, was up-regulated. GFAP is expressed in the central nervous system in astrocytes and is involved in cell communication and the functioning of the blood–brain barrier. However, after neuronal damage, astrocytes infiltrate the damaged area and GFAP levels increase (O’Callaghan and Sriram 2005). Increases in brain GFAP have been documented following exposure to environmental chemicals (Brock and O’Callaghan 1987) and have become an accepted biomarker for neurotoxicity (O’Callaghan and Sriram 2005).

Protein metabolism was heavily influenced by DE-71 exposure in our study. Our data show up-regulation of proteins involved in protein folding (T-complex protein 1 subunit gamma; TCPG) and complex assembly (GRP78). In addition, several proteins important in the brain’s response to stress were affected. In the hippocampus, proteins related to the chaperone/ubiquination pathway were increased by DE-71 (see Supplemental Material, Table S3). Heat shock proteins such as HSP74, HSP7C, and HSP105 were all increased approximately 1.6- to 1.7-fold in DE-71-treated animals compared with vehicle controls (60–70% increase over controls). HSPs are molecular chaperones that have roles in protein synthesis, protect against stress by aiding in refolding of slightly damaged proteins, or can transfer severely damaged proteins to the proteasome for degradation (Becker and Craig 1994). Using IPA, we found several ontology groupings that highlighted protein ubiquination pathways as being significantly impacted in the hippocampus by DE-71 (Figure 3).

Of particular interest, the only two proteins that showed decreased expression in DE-71–exposed rats have roles in immune cell activation. As described above, MARCKS has a role in cytoskeleton rearrangement in response to external stimuli. In addition, induction of MARCKS has been observed following microglial activation, linking it to neurodegenerative processes (Murphy et al. 2003). However, results of the present study indicate a decrease (–1.39-fold) in MARCKS protein. The second down-regulated protein, PSA1 (also known as Macropain; –1.40 fold), in addition to its role in protein degradation as a component of the proteosome, is known to mediate the LPS-induced activation of macrophages with consequent transcription of genes that encode proinflammatory regulators of the immune response (Martínez-Solano et al. 2009). Thus, depletion of these proteins by DE-71 could negatively impact the brain’s capacity to mount a defense against damage and/or insult.

It is important to note that there is considerable crossover in secondary functions for many of the proteins listed. For example, in Supplemental Material, Table S3, the enolases were grouped under “Cell growth and function” because of their roles in carbohydrate metabolism and glycolysis, but they also have roles in stress response in neurons (Díaz-Ramos et al. 2012). Glutamine synthetase is important in the brain for GABA (γ-aminobutyric acid) synthesis and is also a significant player in energy metabolism as part of the citric acid cycle. Proteins with general roles in cell proliferation in the nervous system often have roles in neuronal function. For example, DCTN2 is important in chromosome alignment during mitosis (Quintyne et al. 1999) and may also play a role in neurogenesis and synaptogenesis during brain development or in plasticity following damage. Dynactin is important in linking Sun1/2 and Syne/Nesprin-1/2 to the nuclear membrane; both of these proteins are important to the nuclear movement that is critical for neurogenesis and neuronal migration (Zhang et al. 2009). This process is required for microtubule extension during growth cone remodeling and axonal growth (Grabham et al. 2007). The nature of many of the proteins affected by DE-71 exposure is suggestive of neuronal damage and plasticity related to repair and homeostatic mechanisms.

PDIA3, also known as ERp57, was chosen for our validation experiments because of its multiple functions in the processes identified by the proteomics data set. The expression of PDIA3 protein was up-regulated in both 2D DIGE and Western blot analyses. As a constituent of the endoplasmic reticulum, in conjunction with other chaperone proteins, PDIA3 assists in glycoprotein folding (Bedard et al. 2005), and it also plays a role in synaptic plasticity (Hofmann and Kirsch 2012). A common mode of action could be expected between chemically induced neurotoxicity and neurodegenerative diseases. In this context, it was reported by Erickson et al. (2005) that PDIA3 acts as a carrier protein for β-amyloid and that plaque formation in Alzheimer’s disease may be due to faulty endoplasmic reticulum posttranslational processing. In addition, Wilhelmus et al. (2011) proposed that PDIA3 may be involved in α-synuclein accumulation in Parkinson’s disease.

**Figure 4:**
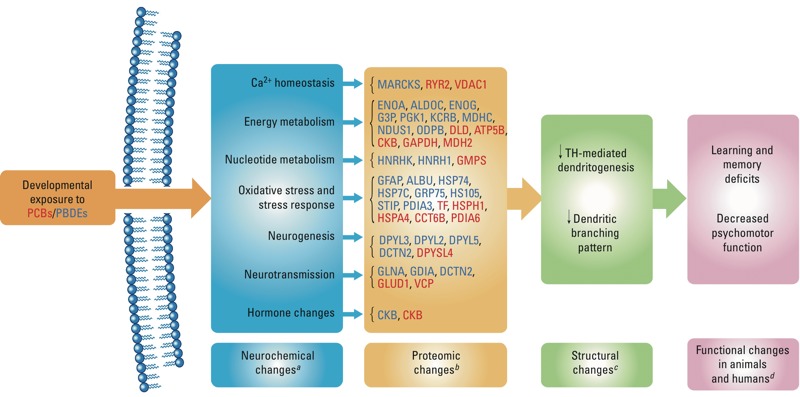
Schematic showing possible adverse outcome pathways for the developmental neurotoxicity of PCBs/PBDEs using a systems biology approach. The schematic highlights the pathways that link PCB/PBDE exposure to neurochemical changes and genomic and proteomic changes that may lead to structural and functional changes. Proteins listed in red were altered by developmental exposure to PCB; those listed in blue were altered by developmental PBDE exposure. Although different proteins were altered by these structurally related chemicals (PBDEs and PCBs), it is very interesting to note that similar pathways were altered by the developmental exposure and hence may have a common mode of action for the adverse effects associated with these chemicals. ***^a^***Data from Kodavanti et al. (2005, 2010). ***^b^***Data from the present study and Kodavanti et al. (2011). ***^c^***Data from Ibhazehiebo et al. (2011). ***^d^***Data from Eriksson et al. (2001), Eskenazi et al. (2013), Herbstman et al. (2010), and Rice et al. (2007).

PBDEs belong to the family of organohalogen chemicals that are ubiquitous around the world, persistent in the environment, and of continued concern in health issues. Previously, our laboratory conducted a similar proteomics study on the PCB mixture Aroclor 1254 (Kodavanti et al. 2011). In experiments with these structurally related compounds, we observed an increase in proteins related to energy metabolism, chaperone function, neuronal plasticity, and neuronal damage, similar to that found with the PBDE mixture in the present study (Figure 4). Disruptions in PKC activation, calcium signaling, oxidative stress, and thyroid hormone perturbations are recognized as adverse outcome pathways for PCB exposure (Figure 4; Kodavanti 2005; Kodavanti et al. 1998). In the present study, MARCKS protein was down-regulated by DE-71. MARCKS is a widely distributed primary substrate for PKC in the cytoplasm and is involved in neurite outgrowth and dendritic spine morphology during development (Calabrese and Halpain 2005). The Aroclor 1254–dependent decrease in PKC could have been due, in part, to a lack of the MARCKS substrate, suggesting that a similar loss in PKC activity could occur after DE-71 exposure, with possible effects on nervous system development (Figure 4). Adverse effects of these two groups of chemicals included both morphometric and functional changes. With Aroclor 1254 exposure, both dendritic growth and branching was decreased following developmental exposure (Lein et al. 2007), along with decreased motor activity and learning and memory deficits (Eriksson and Fredriksson 1996). In agreement with effects of Aroclor 1254, suppression of thyroid hormone–mediated dendritogenesis and dendritic branching pattern (Ibhazehiebo et al. 2011), as well as decreased motor activity and memory deficits, have been reported with PBDE exposure (Herbstman et al. 2010). Thus, we propose common adverse-outcome pathways within this extended family of chemicals (Figure 4). Current proteomic data support the idea that neurotoxic effects observed after developmental DE-71 exposure could arise from the disruption of the normal ontogenetic pattern of nervous system growth and development through perturbation of intracellular signaling pathways, causing oxidative stress and disrupting energy homeostasis.

## Supplemental Material

(521 KB) PDF
