# Supplementary material for: Developmental Exposure to a Commercial PBDE Mixture: Effects on Protein Networks in the Cerebellum and Hippocampus of Rats
Source: Environ Health Perspect. 2014 Dec 19;123(5):428–36. doi: 10.1289/ehp.1408504 (PMC4421769; doi:10.1289/ehp.1408504)
Supplement: (521 KB) PDF [file ehp.1408504.s001.508.pdf]

Environmental Health Perspectives doi:10.1289/ehp.1408504

Supplemental Material

Developmental Exposure to a Commercial PBDE Mixture: Effects on Protein Networks in the Cerebellum and Hippocampus of Rats

Prasada Rao S. Kodavanti, Joyce E. Royland, Cristina Osorio, Witold M. Winnik, Pedro Ortiz, Lei Lei, Ram Ramabhadran, and Oscar Alzate

Table S1. Quantitative proteomic analysis of differentially expressed protein in the rat cerebellum and hippocampus following gestational and developmental exposure to DE-71.

Sample Number	Protein Name	Accession #	pI	MW (kDa)	Protein Score (Mascot)
**CEREBELLUM**					
1C	Heterogeneous nuclear ribonucleoprotein H2	Q6AY09	5.89	49,294	184
2C	Alpha-enolase	P04764	6.16	47,128	551
3C	Fructose-bisphosphate aldolase C	P09117	6.67	39,284	1200
4C	Heterogeneous nuclear ribonucleoprotein A3	Q8BG05	9.10	39,652	285
**HIPPOCAMPUS**					
1H	Heat shock protein 105 kDa	Q66HA8	5.40	96,419	129
2H	Heat shock 70 kDa protein 4	O88600	5.13	94,057	203
3H	Transitional endoplasmic reticulum ATPase	P46462	5.14	89,349	399
4H	Eukaryotic translation initiation factor 4B	Q5RKG9	5.59	69,064	124
5H	NADH-ubiquinone oxidoreductase1.47 75 kDa subunit, mitochondrial	Q66HF1	5.65	79,412	208
6H	Myristoylated alanine-rich C-kinase substrate	P30009	4.32	29,795	229
7H	78 kDa glucose-regulated protein	P06761	5.07	72,347	229
8H	Stress-70 protein, mitochondrial	P48721	5.97	73,858	215
9H	Serum albumin	P02770	6.09	68,731	40
10H	Heat shock cognate 71 kDa protein	P63018	5.37	70,871	320
11H	Neurofilament light polypeptide	P19527	4.63	61,335	212
12H	Dihydropyrimidinase-related protein 5	Q9JHU0	6.60	61,540	474
13H	Dihydropyrimidinase-related protein 5	Q9JHU0	6.60	61,540	90
14H	Dihydropyrimidinase-related protein 2	P47942	5.95	62,278	311
15H	Dihydropyrimidinase-related protein 2	P47942	5.95	62,278	513
16H	T-complex protein 1 subunit gamma	Q6P502	6.23	60,647	79
17H	Stress-induced-phosphoprotein 1	O35814	6.40	62,570	379
18H	Tubulin alpha-1C chain	Q6AYZ1	4.96	49,937	85
19H	Rab GDP dissociation inhibitor alpha	P50398	5.00	50,537	103
20H	Dihydropyrimidinase-related protein 3	Q62952	6.04	61,967	209
21H	Dihydropyrimidinase-related protein 3	Q62952	6.04	61,967	194
22H	T-complex protein 1 subunit epsilon	Q68FQ0	5.51	59,537	149
23H	Protein disulfide-isomerase A3	P11598	5.88	56,623	595
24H	Heterogeneous nuclear ribonucleoprotein H	Q8VHV7	5.70	49,188	100

Sample Number	Protein Name	Accession #	pI	MW (kDa)	Protein Score (Mascot)
25H	Glial fibrillary acidic protein	P47819	5.35	49,957	349
26H	Glial fibrillary acidic protein	P47819	5.35	49,957	300
27H	Alpha-enolase	P04764	6.16	47,128	516
28H	Dynactin subunit 2	Q6AYH5	5.14	44,148	295
29H	Gamma-enolase	P07323	5.03	47,141	599
30H	Creatine kinase B-type	P07335	5.39	42,725	286
31H	Creatine kinase B-type	P07335	5.39	42,725	88
32H	Phosphoglycerate kinase 1	P16617	8.02	44,538	356
33H	Septin-5	Q9JJM9	6.34	42,852	69
34H	Glyceraldehyde-3-phosphate dehydrogenase	P04797	8.14	35,828	100
35H	Glyceraldehyde-3-phosphate dehydrogenase	P04797	8.14	35,828	199
36H	Pyruvate dehydrogenase E1 component subunit beta, mitochondrial	P49432	6.20	38,982	100
37H	Malate dehydrogenase, cytoplasmic	O88989	6.16	36,483	246
38H	Proteasome subunit alpha type-1	P18420	6.14	29,518	80
39H	Glutamine synthetase	P09606	6.64	42,268	188
40H	Glyceraldehyde-3-phosphate dehydrogenase	P04797	8.14	35,828	258
41H	Heat shock 70 kDa protein 4	O88600	5.13	94,057	120
42H	Serum albumin	P02770	6.09	68,731	237
43H	Heterogeneous nuclear ribonucleoprotein K	P61980	5.39	50,944	120
44H	Dihydropyrimidinase-related protein 3	Q62952	6.04	61,967	40
45H	Dihydropyrimidinase-related protein 3	Q62952	6.04	61,967	180
46H	Tubulin alpha-1B chain	Q6P9V9	4.94	50,120	300
47H	Tubulin alpha-1B chain	Q6P9V9	4.94	50,120	300

Table S2. Functional categories of cerebellar proteins identified as significantly different by 2D DIGE analysis at $p \leq 0.05$.

**Function and ref sequence**	**UniProt accession #**	**UniProt entry name**	**Log (ratio)**	**T-test p-value**	**Description**
**Carbohydrate metabolism/ glycolysis**					
NP_001103378.1	P04764	ENOA	1.62	0.046	Alpha-enolase (gene name, *Eno1*); enzyme with roles in glycolysis, growth control and hypoxia tolerance, in neurons interacts with plasminogen and promotes its activation
NP_036629.1	P09117	ALDOC	1.46	0.044	Fructose-bisphosphate aldolase C (gene name, *Aldoc*); axonal component with role in carbohydrate degradation; glycolysis
**Nucleotide metabolism**					
NP_001014041.1	Q6AY09	HNRH2	1.34	0.044	Heterogeneous nuclear ribonucleoprotein H2 (gene name, *Hnrnph2*); component of hnRNP complex involved in processing preRNA to functional RNA, also mediates alternative splicing
NP_444493.1	Q8BG05	ROA3	1.57	0.048	Heterogeneous nuclear ribonucleoprotein A3 (gene name, *Hnrnpa3*); component of hnRNP complex involved in processing preRNA to functional RNA, also mediates alternative splicing

Table S3. Functional categories of hippocampal proteins identified as significantly different by 2D DIGE analysis at $p \leq 0.05$.

**Function and ref sequence**	**UniProt accession #**	**UniProt entry name**	**Log (ratio)**	**T-test p-value**	**Description**
**Cell growth and function**					
Carbohydrate metabolism and glycolysis					
NP_058704.1	P04797	G3P**	2.01/1.62<br>1.52	0.009/0.030<br>0.028	Glyceraldehyde-3-phosphate dehydrogenase (gene name, *Gapdh*); carbohydrate degradation and glycolysis
NP_001103378.1	P04764	ENOA	1.78	0.007	Alpha-enolase (gene name, *Eno1*); enzyme with roles in glycolysis, growth control and hypoxia tolerance, in neurons interacts with plasminogen and promotes its activation
NP_647541.1	P07323	ENOG	1.62	0.007	Gamma-enolase (gene name, *Eno2*); role in carbohydrate degradation and glycolysis, neurotrophic and neuroprotective properties,
NP_445743.2	P16617	PGK1	1.45	0.006	Phophoglycerate kinase 1 (gene name, *Pgk1*); roles in carbohydrate degradation, glycolysis
Cytoskeleton					
NP_001037735.1	Q6P9V9	TBA1B*	1.79/1.43	0.014/0.002	Tubulin alpha-1B chain (gene name, *Tuba1b*); major component of microtubules, cytoskeleton
NP_446383.3	Q9JJM9	SEPT5	1.54	0.017	Septin-5 (gene name, *Sept5*); a cytoskeletal filament forming GTPase, roles in cytokinesis
NP_113971.1	P19527	NFL	1.50	0.002	Neurofilament light polypeptide (gene name, *Nef1*); neuronal cytoskeleton
NP_001011995.1	Q6AYZ1	TBA1C	1.45	0.030	Tubulin alpha-1C chain (gene name, *Tuba1c*); major component of microtubules, cytoskeleton
XP_002729011	P30009	MARCS	-1.39	0.003	Myristoylated alanine-rich C-kinase substrate (gene name, *Marcks*); most prominent substrate for PKC, binds calmodulin, actin and synapsin, actin cross-linking protein
Energy metabolism					
NP_036661.2	P07335	KCRB*	1.84/1.55	0.003/0.036	Creatine kinase B-type (gene name, *Ckb*); catalyzes transfer of phosphate between ATP and other phosphogens, role in energy transduction

Function and ref sequence	UniProt accession #	UniProt entry name	Log (ratio)	T-test p-value	Description
NP_150238.1	O88989	MDHC	1.56	0.002	Malate dehydrogenase, cytoplasmic (gene name, *Mdh1*); part of citric acid cycle, roles in energy metabolism and gluconeogenesis
NP_001005550.1	Q66HF1	NDUS1	1.47	0.001	NADH-unbiquinone oxidoreductase 75 kDa subunit, mitochondrial precursor (gene name, *Ndusf1*); component of Complex I in electron transport
NP_001007621.1	P49432	ODPB	1.34	0.017	Pyruvate dehydrognease E1 component subunit beta, mitochondrial (gene name, *Pdhb*); roles in glycolysis and energy metabolism
Neurotransmission					
NP_058769.4	P09606	GLNA	1.25	0.012	Glutamine synthetase (gene name, *Glu1*); catalyzes the production of glutamine and 4-aminobutanoate (gamma-aminobutyric acid, GABA), roles in protein synthesis and as an energy source as part of the citric acid cycle
NP_058784.2	P50398	GDIA	1.27	0.042	Rab GDP dissociation inhibitor alpha (gene name, *Gdi1*); regulates the GDP/GTP exchange reaction of most Rab proteins, regulates Rab GTPase recycling; plays a role in Rab3a GRPase recycling during neurotransmitter release
Nucleotide metabolism					
NP_476482	P61980	HNRHK	1.38	0.003	Heterogeneous nuclear ribonucleoprotein K (gene name, *Hnrnphk*); component of hnRNP complex involved in processing preRNA to functional RNA, role in maintenance of cellular ATP levels in stress conditions via protection of their target mRNAs
NP_543172.1	Q8VHV7	HNRH1	1.33	0.050	Heterogeneous nuclear ribonucleoprotein H (gene name, *Hnrnph1*); component of hnRNP complex involved in processing preRNA to functional RNA, also mediates alternative splicing
Proliferation/ER transport					
NP_446316.1	P46462	TERA	1.78	0.002	Transitional endoplasmic reticulum ATPase (also called Valosin-containing protein; gene name, *VCP*); necessary for fragmentation of Golgi stacks during mitosis and for their reassembly after mitosis, roles in vesicle budding from ER

Function and ref sequence	UniProt accession #	UniProt entry name	Log (ratio)	T-test p-value	Description
**Plasticity**					
Axonogenesis					
NP_037066.1	Q62952	DPYL3***	2.00/2.00<br>1.97/1.58	0.020/0.004<br>0.002/0.032	Dihydropyrimidinase-related protein 3 (gene name, *Dpysl3*); necessary for semaphorin signaling and cytoskeletal remodeling; roles in axon guidance, growth cone collapse and cell migration
NP_001099187.1	P47942	DPYL2*	1.85/1.85	0.009/0.006	Dihydropyrimidinase-related protein 2 (gene name, *Dpysl2*); necessary for semaphorin signaling and cytoskeletal remodeling; roles in axon guidance, growth cone collapse and cell migration
NP_075412.1	Q9JHU0	DPYL5*	1.86/1.73	0.017/0.005	Dihydropyrimidinase-related protein 5 (gene name, Dpysl5); may have role in neuronal differentiation and/or axonogenesis
Synaptogenesis/proliferation					
NP_001004239.1	Q6AYH5	DCTN2	1.43	0.005	Dynactin subunit 2 (gene name, *Dctn2*); plays a role in chromosome alignment and spindle organization during mitosis, may have role in synaptogenesis
**Protein chemistry**					
Catabolism/anti-inflammatory					
NP_058974.1	P18420	PSA1	-1.40	0.012	Proteasome subunit alpha type-1 (gene name, *Psma1*); also called macropain, a component of the proteosome, mediates the LPS-induced signal, may be involved in the anti-inflammatory response
Complex assembly					
NP_037215.1	P06761	GRP78	1.34	0.036	78 kDa glucose-regulated protein (gene name, *Hspa5*); role in multimeric protein complex assembly
Folding					
NP_954522.1	Q6P502	TCPG	1.38	0.020	T-complex protein 1 subunit gamma (gene name, *Cct3*); molecular chaperone that assists in protein folding, known to play a role in folding of actin and tubulin
NP_001004078.1	Q68FQ0	TCPE	1.46	0.044	T-complex protein 1 subunit epsilon (gene name, *Cct5*); molecular chaperone that assists in protein folding, known to play a role in folding of actin and tubulin

Function and ref sequence	UniProt accession #	UniProt entry name	Log (ratio)	T-test p-value	Description
Synthesis					
NP_001008325	Q5RKG9	Q5RKG9	1.33	0.005	Eukayotic translation initiation factor 4B (gene name, *Eif4b*); protein synthesis
**Stress response**					
Glial cell activation					
NP_058705.2	P47819	GFAP*	1.42/1.41	0.001/0.042	Glial fibrillary acidic protein (gene name, *Gfap*); a class III intermediate filament and astrocyte cell specific marker, biomarker of CNS damage
NP_599153.2	P02770	ALBU*	1.57/1.30	0.025/0.042	Serum Albumin (gene name, *Alb*); serum protein with roles in transport and osmotic regulation, reported to be synthesized by microglia and up-regulated when they are activated
Protein chaparones/ ubiquination pathway					
NP_705893.1	O88600	HSP74*	1.64/1.41	0.009/0.003	Heat shock 70 kDa protein 4 (gene name , *Hspa4*); up-regulated in neuronal cells with ischemia, member of HSP70 family with roles in protecting against protein unfolding
NP_077327.1	P63018	HSP7C	1.71	0.019	Heat shock cognate 71 kDA protein (gene name, *Hspa8*); a transcriptional repressor and chaperone
NP_001094128.1	P48721	GRP75	1.41	0.016	Stress-70 protein, mitochondrial (gene name, *Hspa*9) (also called mortalin) ; roles in cell proliferation and cellular aging, may also act as chaperone
NP_001011901	Q66HA8	HS105	1.71	0.001	Heat shock protein 105 kDA (gene name, *Hsph1*); prevents aggregation of denatured proteins, inhibits HSPA8/ HSC70, ATPase and chaperone activities
NP_620266.1	O35814	STIP1	1.42	0.004	Stress-induced-phosphoprotein 1 (gene name, *Stip1*); mediates association of chaperones HSC70 and HSP90 (HSPCA and HSPCB)
Redox homeostasis					
NP_059015.1	P11598	PDIA3	1.67	0.010	Protein disulfide-isomease A3(also called ERP57; gene name, *Pdia3*); catalyzes the rearrangement of S-S bonds in proteins, possibly inhibited by acidic phospholipids, role in cell redox homeostasis

Function and ref sequence	UniProt accession #	UniProt entry name	Log (ratio)	T-test p-value	Description
**Unidentified**					
Unknown	unknown	spot #1449	1.92	0.028	unknown
Unknown	unknown	spot # 1391	1.87	0.018	unknown
Unknown	unknown	spot # 1144	1.80	0.043	unknown
Unknown	unknown	spot # 879	1.74	0.037	unknown
Unknown	unknown	spot # 963	1.69	0.013	unknown
Unknown	unknown	spot # 1300	1.67	0.038	unknown
Unknown	unknown	spot # 1430	1.62	0.012	unknown
Unknown	unknown	spot #1351	1.57	0.035	unknown
Unknown	unknown	spot #486	1.56	0.001	unknown
Unknown	unknown	spot # 1647	1.56	0.046	unknown
Unknown	unknown	spot # 1121	1.54	0.013	unknown
Unknown	unknown	spot # 918	1.49	0.012	unknown
Unknown	unknown	spot # 1654	1.46	0.036	unknown
Unknown	unknown	spot #887	1.47	0.007	unknown
Unknown	unknown	spot #707	1.46	0.044	unknown
Unknown	unknown	spot #625	1.43	0.038	unknown
Unknown	unknown	spot #777	1.40	0.012	unknown
Unknown	unknown	spot #1207	1.37	0.039	unknown
Unknown	unknown	Spot #532	1.30	0.005	unknown
Unknown	unknown	spot # 1394	1.34	0.043	unknown
Unknown	unknown	spot #1442	1.13	0.048	unknown
Unknown	unknown	spot #714	-1.48	0.013	unknown
Unknown	unknown	spot #1652	-2.15	0.021	unknown

* duplicate spots; ** triplicate spots; *** quadruplicate spots

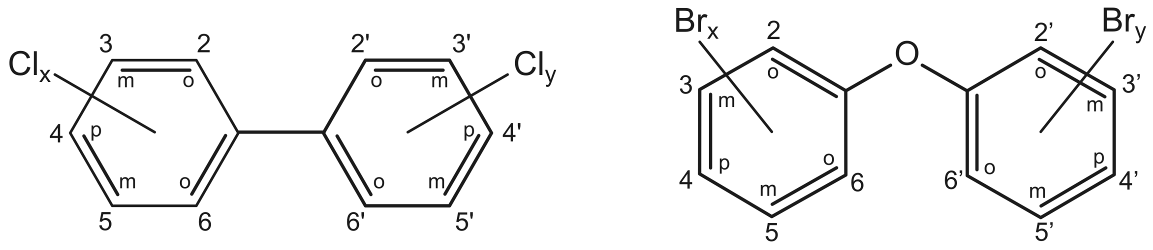

PCBs PBDEs

Figure S1

Figure S1. Structural features of polychlorinated biphenyls (PCBs) and polybrominated diphenyl ethers (PBDEs). The letters (o), (m), and (p) indicate *ortho*, *meta*, and *para* substitutions for chlorines in the case of PCBs or bromines in the case of PBDEs. The numbers indicate position of chlorines while x or y indicates the number of chlorines or bromines.

A

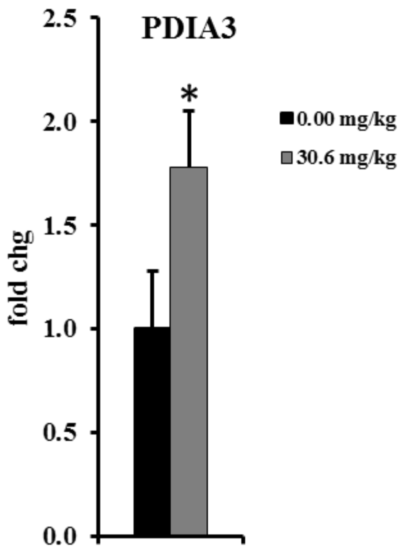

B

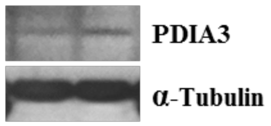

Figure S2

Figure S2. Western blot analysis of the protein PDIA3 (also called as ERP57) used to confirm the 2D DIGE analysis. Panel A shows the relative fold change in expression of hippocampal proteins of DE-71-treated rat pups compared to controls (n = 3). Panel B shows a corresponding representative example of the level of expression in the gel of PDIA3 and the internal standard, α-tubulin. Error bars indicate mean ± SEM of three independent determinations (* different at $p < 0.05$).
